# The structure of INI1/hSNF5 RPT1 and its interactions with the c‐MYC:MAX heterodimer provide insights into the interplay between MYC and the SWI/SNF chromatin remodeling complex

**DOI:** 10.1111/febs.14660

**Published:** 2018-10-01

**Authors:** Susan Sammak, Mark D. Allen, Najoua Hamdani, Mark Bycroft, Giovanna Zinzalla

**Affiliations:** ^1^ Microbiology, Tumor and Cell Biology (MTC) Department Karolinska Institutet Stockholm Sweden; ^2^ MRC Laboratory of Molecular Biology Cambridge UK

**Keywords:** BAF/PBAF complexes, INI1/hSNF5/BAF47/SMARCB1 subunit, MYC, protein–protein interactions, transcription factors

## Abstract

c‐MYC and the SWI/SNF chromatin remodeling complex act as master regulators of transcription, and play a key role in human cancer. Although they are known to interact, the molecular details of their interaction are lacking. We have determined the structure of the RPT1 region of the INI1/hSNF5/BAF47/SMARCB1 subunit of the SWI/SNF complex that acts as a c‐MYC‐binding domain, and have localized the interaction regions on both INI1 and on the c‐MYC:MAX heterodimer. c‐MYC interacts with a highly conserved groove on INI1, while INI1 binds to the c‐MYC helix‐loop‐helix region. The binding site overlaps with the c‐MYC DNA‐binding region, and we show that binding of INI1 and E‐box DNA to c‐MYC:MAX are mutually exclusive.

**Abbreviations**
BAF: BRG1‐ or HBRM‐associated factors
c‐MYC: avian myelocytomatosis virus oncogene cellular homolog
HSQC: heteronuclear single quantum coherence
INI1: integrase interactor 1
MAX: MYC‐associated factor X
MIZ1: MYC‐interacting zinc finger protein‐1
PPI: protein–protein interaction
SMARCB1: SWI/SNF related, matrix associated, actin dependent regulator of chromatin, subfamily B, member 1
SWI/SNF: SWItch/sucrose nonfermentable

## Introduction

The transcription factor c‐MYC (hereafter MYC) acts as a master regulator of genes involved in cell growth, differentiation, metabolism, and apoptosis. Deregulated expression of MYC occurs in the majority of human cancers, playing a pivotal role in tumorigenesis and cancer progression 1, 2. In mouse models, inactivation of MYC dramatically halts tumor cell growth and proliferation, without invoking tumor escape pathways 1, 2, making targeting MYC an attractive approach for anticancer therapy 3. MYC is also an emerging target in other areas of human diseases, such as inflammation and heart disease 4, 5. As MYC dramatically increases the efficiency of somatic cell reprogramming, modulating MYC functions is also important in regenerative medicine 6. Although MYC physiology and pathology have been extensively studied, we still do not know how MYC works at the molecular level, which is a key to be able to target it pharmacologically. MYC coordinates the expression of a large, extremely diverse set of genes in a highly context‐dependent manner. MYC operates within a network of protein–protein interactions (PPIs), crucial for both directing MYC to specific genomic sites and for modulating gene expression 7, 8. However, our knowledge of these interactions is limited.

Several studies have shown that the SWI/SNF chromatin remodeling complex is part of the MYC interactome 9, 10, 11, 12. This multiprotein complex 13 uses ATP to alter chromatin structure by repositioning nucleosomes 13 and plays a key role in regulating gene expression during cell differentiation. Mutations that inactivate SWI/SNF subunits are found in around 20% of human cancers 14. Its role in cancer is complex as it has reported that depending on the type of the tumor the SWI/SNF complex can either inhibit tumor progression, or be required for cancer cell growth. Targeting the SWI/SNF complex is, thus, paradoxically emerging as a potential strategy for anticancer therapy 15.

The interplay between MYC and the SWI/SNF chromatin remodeling complex seems to be multifaceted and very much context‐ and gene dependent 16, 17, 18, 19, 20.

The INI1 (syn hSNF5, BAF47, SMARCB1) subunit 10, 21, 22 has been shown to play a major role in the interaction of the SWI/SNF complex with MYC. This subunit has also been implicated in the recruitment of other transcription factors 23, other chromatin‐associated proteins and a number of viral proteins, such as HIV integrase 24 and EPV EBNA2 25. INI1 was the first subunit of this complex identified to be mutated in cancer. Nonsense mutations and deletions that abolish INI1 expression are present in rhabdoid tumors, the most common malignant CNS tumors of children below 6 months of age. Analysis of the genomes of rhabdoid tumors shows very few other mutational events indicating that epigenetic dysregulation is the central mechanism of oncogenesis 26.

In rhabdoid tumor‐derived cells reintroduction of INI1 appears to suppress MYC functions. In other cancer cell lines, it has been shown instead that INI1 contributes to MYC transcriptional activity 21, and that overexpression of the region of INI1 that binds to MYC blocks MYC transcriptional activation 27.

We set out to determine how these two important transcription regulators interact at the molecular level and have revealed a complex network of competing interactions.

## Results

### Structure determination of INI1/hSNF5 RPT1

**Figure 1:**
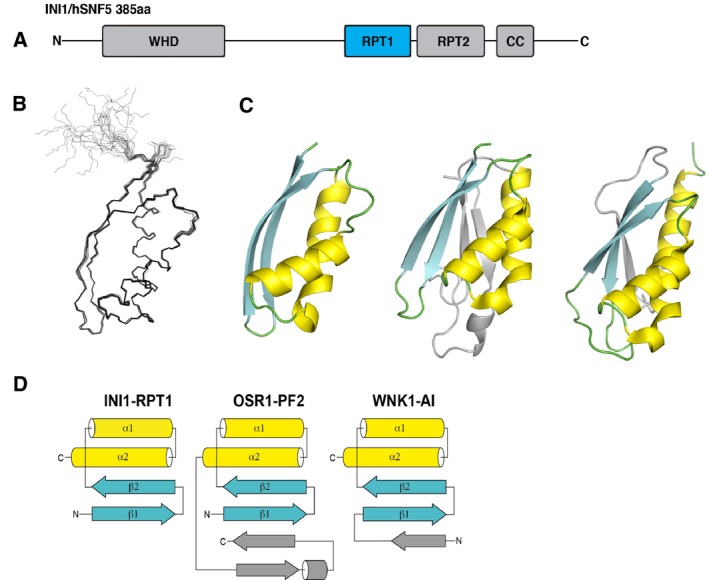
Structure of INI1/hSNF5 RPT1. (A) Representation of the domain structure of INI1. (B) Overlay of the 20 lowest energy NMR structures of RPT1. (C) From left to right: cartoon representations of the X‐ray structure of RPT1, and the structures of the OSR1 CCT domain and the autoinhibitory domain of WNK1 (WNK1‐AI). (D) Schematic of the topologies of the INI1/hSNF5 RPT1, OSR1 CCT, and WNK1‐AI domains.

INI1 is a modular protein consisting of an N‐terminal winged‐helix domain 28 followed by two 60‐amino acid imperfect repeats Repeat 1 (RPT1) and Repeat 2 (RPT2), and a C‐terminal coil–coil domain (Fig. 1A). RPT1 has been shown to be required for MYC interaction 21, 27. The expression of residues 184–258 of INI1, which encompasses RPT1, in *Escherichia coli* provided soluble protein that gave good quality ^1^H,^15^N heteronuclear single quantum coherence (HSQC) spectra with chemical shift dispersion typical of a well‐folded protein. INI1 RPT1 behaved as a monomer by NMR and size exclusion chomatography–multi‐angle laser light scattering (SEC‐MALS) analysis. Protein samples were sufficiently stable to allow the collection of the triple‐resonance and nuclear Overhauser enhancement spectroscopy data needed for chemical shift assignment and solution structure determination (Table 1). The NMR solution structure of this fragment was determined (Fig. 1B) (PDB: 5L7B) and based on this a shorter protein construct (184–252) was expressed that readily crystallized. The crystal structure of this protein was also determined using the NMR structure as a molecular replacement model (Fig. 1C and see also Table 2) (PDB: 5L7A). Residues 185–248 of INI1 form a compact folded domain that contains two antiparallel β‐strands linked by a short loop followed by two α‐helices (Fig. 1D). The two strands form a curved base onto which both helices pack and the structure is capped at either end by loops that link the two strands, and strand 2 and helix 1. This topology places the N‐ and C termini of the domain in close proximity. The fold is stabilized by a hydrophobic core that is primarily formed by residues in β1 (I187, I189, L191, M193, I195) and α2 (F233, I237, I241, I245). In addition, the side chain of Q244 in α2 forms hydrogen bonds to the backbone NH and CO groups of R190 in β1, thereby linking the N‐ and C termini of the domain. Comparison of the INI1 RPT1 structure to other known structures using the program dali 29 reveals significant similarities to domains in two kinases involved in the regulation of osmotic stress, the CCT domain of oxidative stress responsive kinase 1 (OSR1) 30 (Fig. 1C,D) and the autoinhibition domain of lysine‐deficient protein kinase 1 (WNK1) 31 (Fig. 1C,D). Both domains share a core ααββ motif with INI1 RPT1 with additional elements of secondary structure at the periphery of their folds (Fig. 1D). The ααββ motifs of the OSR1 and WNK1 domains can be superimposed onto the structure of INI1 RPT1 with an RMSD of 1.9 and 2.0 Å, respectively.

**Table 1:** Summary of conformational constraints and statistics for the 20 accepted NMR structures of the human INI1/SNF5 RPT1 domain

Structural constraints	
Intraresidue	663
Sequential	393
Medium‐range (2 < |*i* − *j*| < 4)	421
Long‐range (|*i* − *j*| > 4)	539
Dihedral angle constraints	21
TALOS constraints^a^	124
Distance constraints for 38 hydrogen bonds	76
Total	2237
Statistics for accepted structures	
Statistical parameters (±SD)	
RMS deviation for distance constraints	0.0070 ± 0.0004 Å
RMS deviation for dihedral constraints	0.342 ± 0.024°
Mean CNS energy term (kcal·mol^−1^ ± SD)	
*E* (overall)	80.61 ± 4.31
*E* (van der Waals)	19.87 ± 1.35
*E* (distance constraints)	7.69 ± 0.91
*E* (dihedral and TALOS constraints)	2.07 ± 0.28
RMS deviations from the ideal geometry (SD)	
Bond lengths	0.0015 ± 0.0001 Å
Bond angles	0.356 ± 0.0078°
Improper angles	0.250 ± 0.009°
Average atomic RMSD from the mean structure (SD)	
Residues 184–249 (backbone)^b^	0.237 ± 0.053 Å
Residues 184–249 (all heavy atoms)^c^	0.720 ± 0.062 Å
Structural quality^d^	
Residues in most favored region of Ramachanran Plot	88.4%
Residues in additional allowed region of Ramachandran Plot	10.4%
Residues in generously allowed region of Ramachandran Plot	1.2%
Residues in disallowed region of Ramachandran Plot	0.0%
PDB code	5L7B

^a^ Dihedral angles are estimated using TALOS+‐based chemical shifts of backbone atoms of each amino acid. ^b^ Backbone heavy atoms include backbone N, Ca, CO. ^c^ Heavy atoms include both backbone and side chain non‐hydrogen atoms. ^d^ Statistics are for residues 184–259.

**Table 2:** Data collection and refinement statistics

	Native dataset
Data collection	
Space group	P2_1_
Cell dimensions	
*a*,* b*,* c* (Å)	43.619, 73.653, 46.460
α, β, γ (°)	90, 106.596, 90
Resolution (Å)^a^	44.5–2.1 (2.22–2.10)
Total reflections	60 325
Unique reflections	15 711
*R*_sym_ or *R*_merge_ (%)^b^	0.060 (0.438)
*I*/σ*I*	14.6 (3.3)
Completeness (%)	95.6 (93.3)
Redundancy	3.8 (3.9)
Refinement	
Resolution (Å)	45.0–2.1
No. reflections	15 656
*R*_work_/*R*_free_ c	20.0, 0.26.0
Number of atoms	
Protein	2117
Water	130
*B*‐factors	
Protein	34.26
Water	35.40
RMS deviations^d^	
Bond lengths (Å)	0.008
Bond angles (°)	0.918
PDB code	5L7A

^a^ Highest resolution shell is shown in parenthesis. ^b^ *R*_m_:∑*h*∑*i*|I/(*h*,* i*) − I(*h*)|/∑*h*∑*i*/*I*(*h*,* i*) where *I*(*h*,* i*) are symmetry‐related intensities and *I*(*h*) is the mean intensity of the reflection with unique index *h*. ^c^ *R*‐factor = Σ(|*F*_obs_| − *k*|*F*_calc_|)/Σ|*F*_obs_| and *R*‐free is the *R* value for a test set of reflections consisting of a random 5% of the diffraction data not used in refinement. ^d^RMS deviations from ideal geometry for bond lengths and restraint angles (Engh and Huber).

### The MYC:MAX bHLHZip dimer interacts with a conserved site on INI1/hSNF5 RPT1

**Figure 2:**
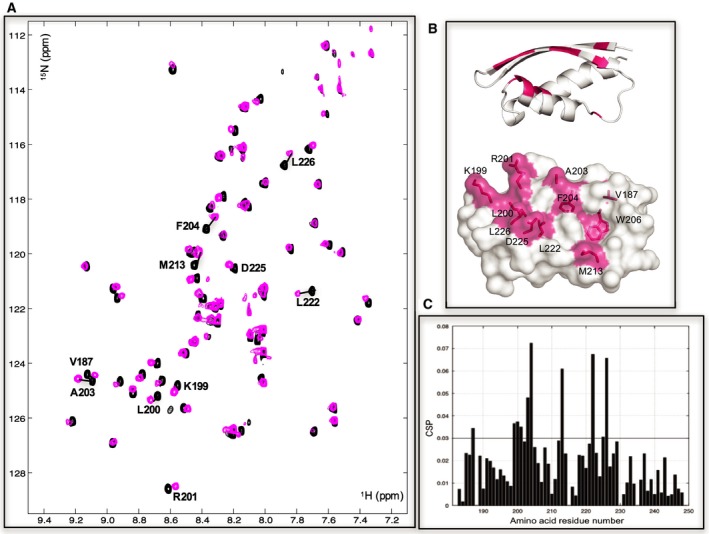
The MYC:MAX bHLHZip dimer binds to a conserved pocket on INI1/hSNF5 RPT1. (A) ^1^H,^15^N HSQC spectra of RPT1 without (black) and with (magenta) the addition of unlabeled MYC:MAX bHLHZip dimer (ratio 1 : 2). Residues undergoing chemical shift changes more than the standard deviation (SD) are labeled in black. (B) Cartoon (top) and molecular surface representation (bottom) of RPT1 showing the residues that undergo chemical shift changes more than the standard deviation are highlighted in magenta and labeled. The chemical shift changes of W206 are for the aromatic proton of the side chain (changes in chemical shifts of the backbone are below the threshold). (C) Diagram showing the differences in chemical shifts induced by binding of the MYC:MAX dimer to the ^15^N‐labeled INI1 RPT1. The black line indicates the calculated standard deviation.

**Figure 3:**
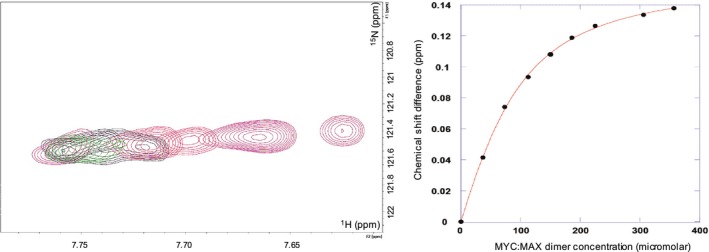
*K*_d_ determination of the binding of the MYC:MAX dimer to the INI1/hSNF5 RPT1. On the left overlay of 15N HSQC spectra showing the variation of chemical shift changes induced on L222 by the addition of the unlabeled MYC:MAX dimer. The peak moves from right to left with increasing concentration of the dimer; on the right, plot of the induced chemical shifts versus dimer concentration, fitted to a single‐site binding curve. INI1 RPT1 was employed at a concentration of 147 μm.

We next set out to identify which region of INI1 RPT1 interacts with MYC. The C‐terminal part of MYC containing the basic helix‐loop‐helix (bHLH) and leucine zipper (Zip) domains has been shown to be required for interaction with INI1 21 and this interaction has been reported to occur with MYC bound to its heterodimerization‐binding partner MAX 22. We therefore coexpressed the bHLHZip region of MYC with the corresponding region (bHLHZip) of MAX. We then titrated purified unlabeled MYC:MAX bHLHZip dimer into ^15^N‐labeled INI1 RPT1. This produced significant changes in the ^1^H,^15^N HSQC spectrum of INI1 RPT1 (Fig. 2C,D). The bound and free forms of the domain are in fast exchange on the NMR timescale, and analysis of the dependence of the changes in chemical shifts induced on the amount of MYC:MAX dimer added gave a *K*_d_ of 44 ± 6 μm for the interaction (Fig. 3). The residues that undergo significant chemical shift changes map to an elongated solvent‐exposed groove between β2 and α1 that has properties typical of a PPI interface (Fig. 2B). Within this binding region, there are a number of solvent‐exposed hydrophobic residues (F204, F218, I221, L226), which form two shallow pockets. The binding interface also contains two charged residues that undergo large changes in chemical shift upon binding: D202, which is partially buried, and D225, which is at the edge of the pocket (Fig. 2). The second strand of the sheet (β2) runs along the edge of binding interface. The backbone amides of β2 (i.e., K199, R201, and A203) all undergo significant changes in chemical shift. The resonance of N207 in the loop between β2 and α1 at one end of the binding site changes intensity rather than changing chemical shift upon binding. This suggests that the interaction with the MYC:MAX dimer may affect the dynamic properties of this region of the protein.

**Figure 4:**
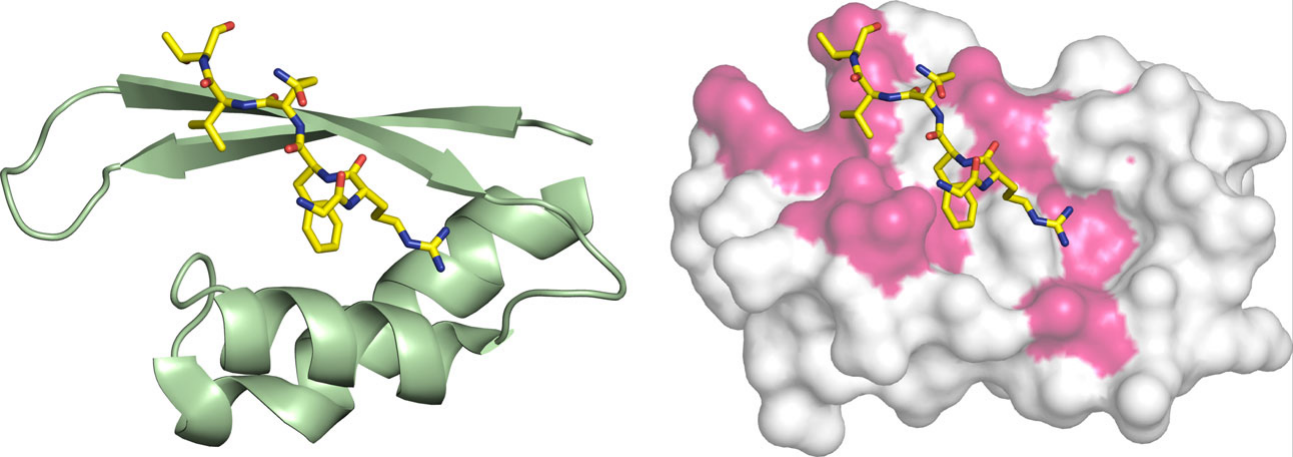
Comparison of the binding site in INI1/hSNF5 RPT1 and OSR1. On the left cartoon representation of the structure of the OSR1 CCT domain (green) in complex with the RFQV‐peptide (yellow). On the right superimposition of the OSR1 CCT domain (not shown) complex with the RFQV‐peptide (yellow) with the RPT1 structure shown as a molecular surface, with the residues that change chemical shifts upon binding to the MYC:MAX complex highlighted in magenta.

The domains structurally similar to RPT1 present in the kinases OSR1 and WNK1 mediate PPIs involved in substrate recognition and regulation. An X‐ray structure of the OSR1 CCT domain bound to a peptide from a protein that regulates its activity has been determined (PDB: 2V3S). The peptide binds in an extended shallow pocket that is equivalent to the binding site identified in INI1 RPT1 (Fig. 4). The MYC:MAX dimer therefore appears to bind to a PPI site common to this family of proteins.

**Figure 5:**
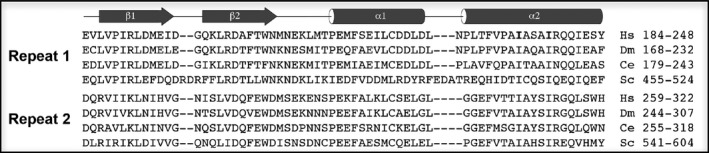
Sequence alignments of the repeats of family members of the INI1/SNF5. At the top, carton representation of the secondary structure elements of RPT1, followed by sequence alignments of RPT1 (middle) and RPT2 (bottom) for the *Homo sapiens* (Hs), *Drosophila melanogaster* (Dm), *Caenorhabditis elegans* (Ce) and *Saccharomyces cerevisiae* (Sc) proteins.

**Figure 6:**
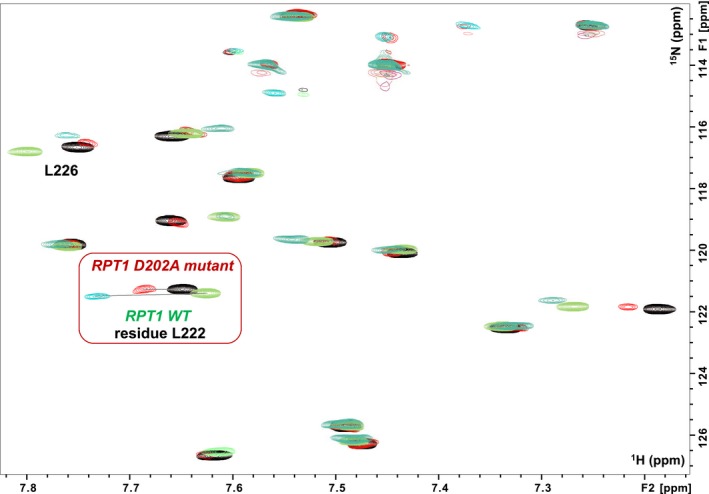
Binding of the MYC:MAX dimer to the INI1 D202A‐mutant. Overlay of a region of the ^1^H,^15^N HSQC spectra of RPT1 INI1 WT without (blue gray) and with (green) unlabeled MYC:MAX bHLHZip dimer (ratio 1 : 1), overlaid with the overlay of the same region of the ^1^H,^15^N HSQC spectra of RPT1 INI1 D202A‐mutant without (black) and with (red) unlabeled MYC:MAX bHLHZip dimer (ratio 1 : 1). Highlighted in the red square is the residue L222 which undergoes the largest change in chemical shift upon binding of MYC:MAX complex to both INI1 RPT1 WT and the D202A mutant. Comparison of the same ratio (1 : 1) illustrates the lower affinity of the DA mutant as the change in chemical shift of L222 is significantly less.

The sequence of RPT1 is identical in species ranging from humans to chicken (Fig. 5). The sequences of INI1 homologs in lower eukaryotes are more diverse, although some residues are highly conserved. These include amino acids that form the hydrophobic core as well as Q244, which, as discussed above, also appears to contribute to the stability of the fold. Many of the residues within the binding sites are also highly conserved including the two aspartates and several of the solvent‐exposed hydrophobic residues. In contrast, solvent‐exposed residues on the face opposite to the binding groove are not conserved. The role of residues in the binding site is difficult to probe by mutagenesis as many of them are involved in structurally important hydrogen bonding, or are hydrophobic residues that also contribute to the stability of the fold. We tested the effect of mutating two hydrophilic residues within the binding site, D202 and N207 to alanine. The integrity of the mutant proteins was assessed by recording HSQC spectra. Only small changes were observed for D202A mutant, however, much larger changes were observed for the asparagine mutant suggesting that its mutation alters the structure of the protein. The aspartate mutant showed reduced affinity to the MYC:MAX dimer, such that it was not possible to accurately determine a dissociation constant for its interaction, thus confirming that it forms part of the binding site (Fig. 6). Repeats 1 and 2 present a high degree of sequence similarity (Fig. 5). Many of the residues that stabilize the fold in RPT1 are also conserved in RPT2, which indicates that the structure of the two repeats is very similar. Many of the residues within the binding site identified in RPT1 are also conserved in RPT2, suggesting that this region is functionally important in RPT2. However, some of the amino acids conserved within the binding site of RPT2 differ from RPT1, which is consistent with data showing that the two repeats have different binding specificities 32.

### The INI1/hSNF5 RPT1 interacts with the helix‐loop‐helix region of the MYC:MAX dimer

We next set out to use NMR to map the region of the MYC:MAX dimer that interacts with INI1 RPT1. Assignments for human MYC:MAX were not available so we expressed ^15^N,^13^C‐deuterated MYC:MAX bHLHZip dimer and obtained backbone assignments for it using triple resonance NMR experiments (BMRB accession number 27571). Complete assignments were obtained for the leucine zipper (Zip), helix 2 (H2), the loop region and the C‐terminal part of helix 1 (H1) of both MYC and MAX. As was observed for v‐MYC 33 the assignments for the N‐terminal part of H1 in both proteins could not be obtained potentially because of exchange broadening due to fraying of this region. The residues from the basic region of both MYC and MAX are poorly dispersed (causing significant overlap in the central region of spectra), which indicates that they lack a stable structure in the absence of DNA, hampering the full assignment of these regions.

**Figure 7:**
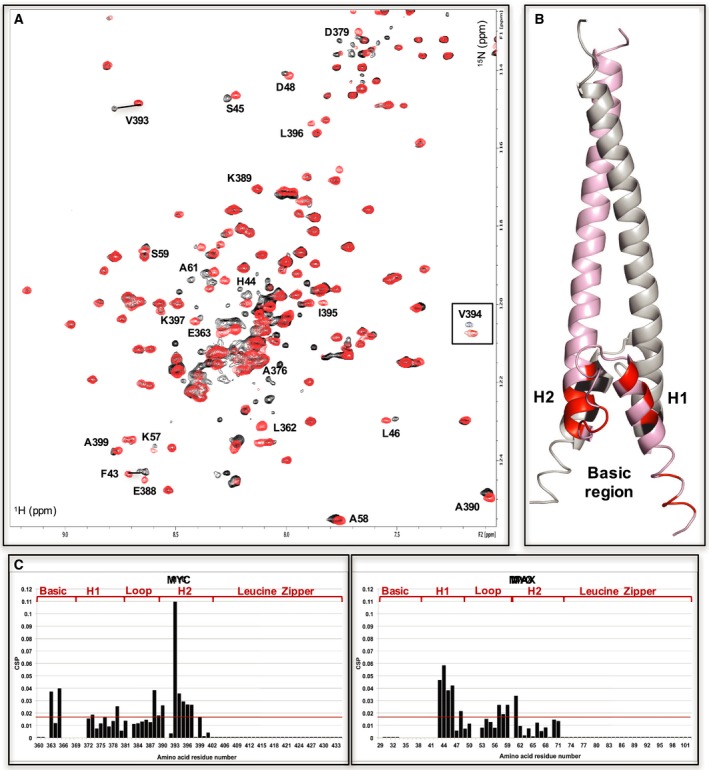
INI1/hSNF5 RPT1 binds to the helix‐loop‐helix region of MYC in the MYC:MAX bHLHZip dimer. (A) ^1^H,^15^N BST TROSY spectra of ^15^N‐labeled MYC:MAX bHLHZip dimer without (red) and with (black, ration 1 : 1) binding to RPT1 that move more than the standard deviation are labeled. The region corresponding to residue V394 is inserted in the spectra in a box to better illustrate the changes of chemical shifts that occur upon binding to INI1. (B) Cartoon representation of the MYC:MAX dimer (PDB =  1NKP, pink: MYC, gray: MAX) with highlighted in red (MYC) and black (MAX) the residues labeled in the spectra. H1 = MYC Helix 1; H2 = MYC Helix 2. (C) Diagrams showing the differences in chemical shifts induced by binding of RPT1 to the ^15^N‐labeled MYC:MAX dimer (MYC on the left, MAX on the right). Residues 29, 34–42, 52, 60, from MAX, and residues 366–71 from MYC are not assigned. L362 is assigned in the free form, but it could not be assigned in the bound form. Residues 51, 382, 391 are prolines. The red line indicates the standard deviation. H1 = MYC Helix 1; H2 = MYC Helix 2.

The addition of the ^1^H,^15^N MYC:MAX bHLHZip dimer to unlabeled INI1 RPT1 produced changes in the chemical shifts of residues in both MYC and MAX (Fig. 7A,C). Several peaks also undergo significant change in intensity, either decreasing or increasing, to the extent that some peaks are only visible in the bound form, which suggests that binding is accompanied by changes of the dynamic/conformational properties of the heterodimer. The largest changes cluster on a discrete patch on MYC, located at one end of the helix‐loop‐helix (HLH) region (Fig. 7B,C), indicating that this is the main binding site of INI1 RPT1 on the dimer complex. The biggest change overall in chemical shift is on MYC at the start of H2 (i.e., V393) (Fig. 7C).

Other significant changes in MYC are in the loop that links H1 and H2, other residues at the N terminus of H2, and residues in the basic region (Fig. 7B,C). Some changes can be seen also in H1 (Fig. 7B,C): these changes are from residues that are close in space to the residues affected in H2 and the Loop.

In MAX, the most significant changes in chemical shift are for residues in H1 (Fig. 7B,C) that are across the heterodimer interface from the affected residues on MYC in H2 (Fig. 7B). There are also a few small changes observed at the start of MAX H2. In both proteins, H1 is only partially folded in the free dimer and these changes may result from tightening of the dimer upon forming the complex, rather than a direct effect of the binding to INI1. The chemical shift mapping results are, therefore, consistent with MYC being the main interaction partner of INI1 RPT1.

**Figure 8:**
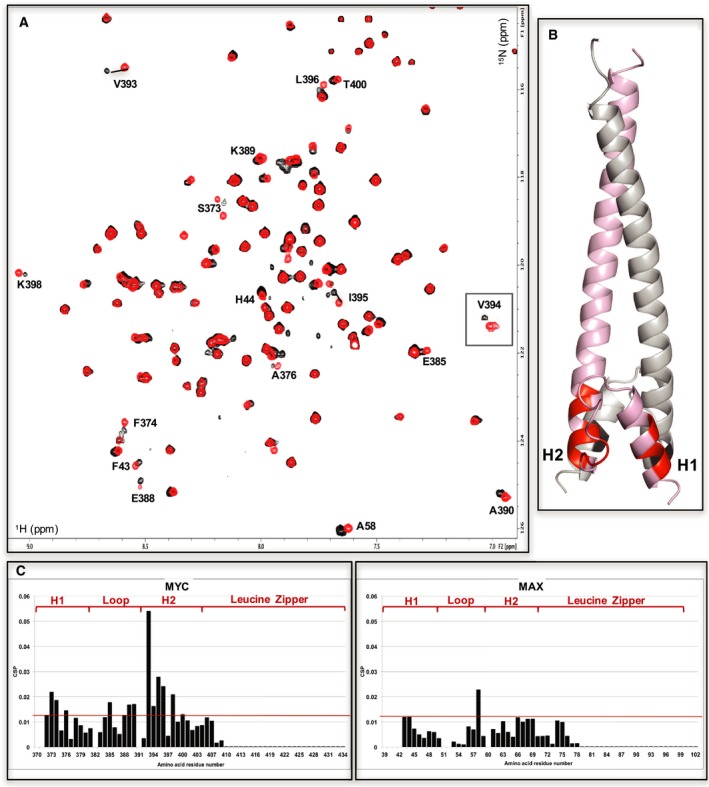
INI1/hSNF5 RPT1 binds to the helix‐loop‐helix region of MYC in the MYC:MAX dimer lacking the basic region. (A) ^1^H,^15^N BST TROSY spectra of ^15^N‐labeled MYC:MAX HLHZip dimer without (red) and with (black, ration 1 : 2) the addition of unlabeled RPT1 (residue 394 is inserted into the spectra as in Fig. 7). Residues implicated in the binding to RPT1 that move more than the standard deviation are labeled. (B) Cartoon representation of the MYC:MAX dimer (pink: MYC, gray: MAX) with highlighted in red (MYC) and black (MAX) the residues labeled in the spectra. H1 = MYC Helix 1; H2 = MYC Helix 2. (C) Diagrams showing the differences in chemical shifts induced by binding of RPT1 to the ^15^N‐labeled MYC:MAX dimer (MYC on the left, MAX on the right). Residues 39–42, 52, 370–71 are not assigned. Residues 51, 382, 391 are prolines. The red line indicates the standard deviation. H1 = MYC Helix 1; H2 = MYC Helix 2.

There are also changes in the basic region of MYC (no changes are observed in the basic region of MAX). Studies on the bHLH‐transcription factors have shown that binding to the HLH region can allosterically induce conformational changes in the basic region 34. Therefore, to understand if the changes in the basic region of MYC are the results of direct binding we then examined the interaction of INI1 with the MYC:MAX HLHZip complex where both basic regions have been removed. As the basic regions in the free MYC:MAX complex are in a disordered conformation removing them also helps with the analysis of the NMR spectra (Fig. 8A). NMR data showed that removing the basic regions does not compromise, or alter the heterodimerization binding interface (Fig. 8A), and biophysical analysis (i.e., DSC) of the HLHZip complex also confirmed that the removal of the basic regions does not destabilize the complex. On the contrary, this complex seems to be more stable having a melting point 7 °C higher than the construct with the basic regions. The NMR spectra of the binding of ^15^N‐labeled MYC:MAX HLHZip complex to unlabeled INI1 RPT1 showed a comparable footprint on the HLH region of MYC to that observed for binding to the MYC:MAX bHLHZip (Fig. 8B,C). Less chemical shift changes can be seen on MAX possibly (Fig. 8C), because as the structure is more stable, there is less consolidation of heterodimer complex upon binding of INI1. The binding affinity, however, is significantly reduced to the point that is not possible to determine a *K*_d_. This suggests that the chemical shift changes observed in the basic region are due to a direct interaction.

The chemical shifts mapping results indicates that INI1 docks onto one face of the heterodimer at one end of the HLH motif of MYC (Fig. 7). This interaction region on MYC does not appear to be an extended linear motif, in contrast to the interaction between the OSR1 CCT domain and the RFQV‐peptide.

### INI1 RPT1 binding to MYC:MAX bHLHZip dimer is incompatible with binding to E‐box DNA

**Figure 9:**
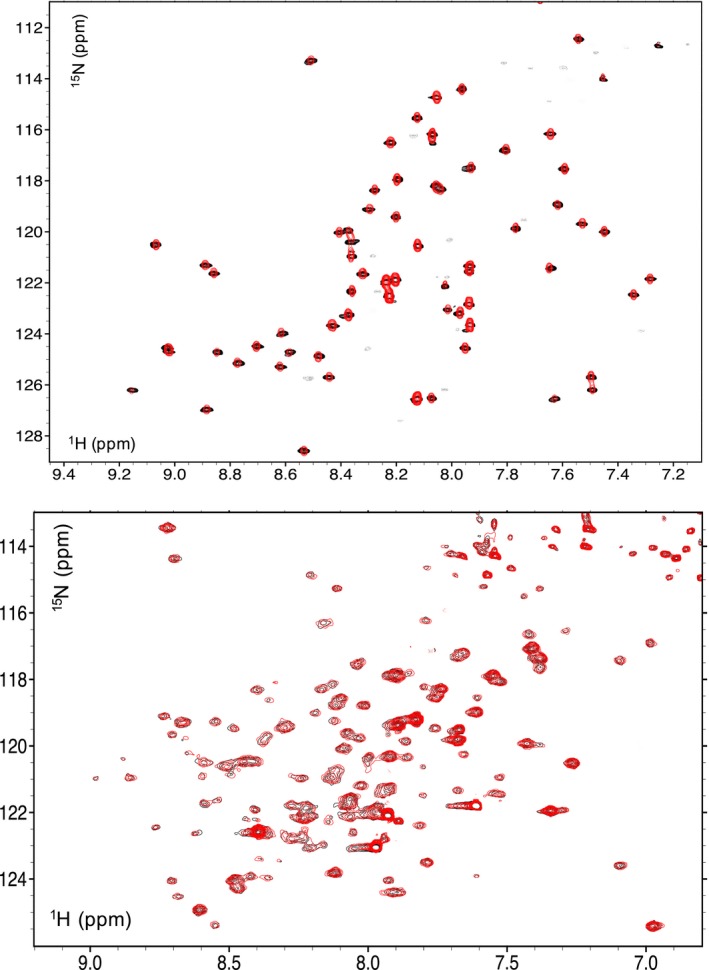
Binding of INI1/hSNF5 RPT1 and DNA to the MYC:MAX bHLHZip dimer are mutually exclusive. Top: overlay of ^1^H,^15^N HSQC spectra of RPT1 without (black) and with (red, ratio 1 : 1) unlabeled MYC:MAX bHLHZip dimer bound to DNA. Bottom: overlay ^1^H,^15^N BST TROSY spectra of ^15^N‐labeled MYC:MAX bHLHZip dimer bound to DNA without (black) and with (red, ratio 1 : 1) unlabeled RPT1 INI1. In both spectra, no chemical shift changes are observed, illustrating that INI1 RPT1 does not bind to the MYC:MAX dimer when this is bound to E‐box DNA.

The MYC:MAX heterodimer binds to canonical E‐box DNA primarily via the basic regions with an affinity in the nanomolar range 35. INI1 RPT1 binds to a region on MYC that is contiguous to the DNA binding motif, and several of the residues in the putative interaction‐site in the HLH motif make contacts with the DNA in the crystal structure of c‐MYC:MAX bound to E‐box DNA (PDB: 1NKP). Furthermore, there is a strong indication that residues in the basic region itself are involved in the binding to INI1. We, therefore, tested if INI1 RPT1 binding is compatible with the binding of the MYC:MAX bHLHZip complex to canonical E‐box DNA. To this end, we first evaluated the binding of a oligonucleotide containing a canonical E‐box sequence (the same E‐box DNA sequence used in the reported crystal structure) by adding ^15^N‐labeled MYC:MAX bHLHZip to the DNA. This produced large changes in chemical shift and line broadening, confirming the formation of the MYC:MAX bHLHZip/DNA complex (MW = 34 kDa). An ITC analysis confirmed that the binding is in the nano‐molar range as previously reported 35. Then, we added ^15^N‐labeled INI1 RPT1 to the unlabeled MYC:MAX bHLHZip/DNA complex. No changes in chemical shifts, or line broadening, were observed in the spectra of the ^15^N‐labeled INI1 RPT1 (Fig 9: top panel). We also added the DNA to the pre‐formed MYC:MAX/^15^N‐labeled INI1 RPT1 complex, and observed displacement of the ^15^N‐labeled INI1 RPT1 by the DNA, as observed by the recovery of a spectrum corresponding to free ^15^N‐labeled INI1 RPT1. No changes in chemical shifts were also observed in the spectra of ^15^N‐labeled MYC:MAX complexed with E‐box DNA when unlabeled INI1 was added (Fig. 9: bottom panel).

## Discussion

Recently several studies have shown that loss of PPIs that are mediated by INI1 is one of the major factors contributing to oncogenesis in malignant rhabdoid tumors 36, 37. Most of the PPIs made by this subunit have been mapped to the repeats (1 and 2) 23, and consistent with this the structure of RPT1 reveals a fold that is an established PPI motif. We have shown that MYC interacts with the canonical binding site of this motif and, therefore, is likely to compete with other binding partners of the SWI/SNF complex. Interestingly, a recent paper has suggested that when MYC is overexpressed it could compete with prodifferentiation transcription factors for binding to the SWI/SNF complex 38. Furthermore, while this study was being completed Yan *et al*. 39. reported that the MYC‐binding groove of RPT1 interacts with the BAF155 subunit of the SWI/SNF complex. This suggests that MYC and other molecules binding to this site could modulate the interactions between SWI/SNF subunits, and alter the activity of the chromatin remodeling complex.

**Figure 10:**
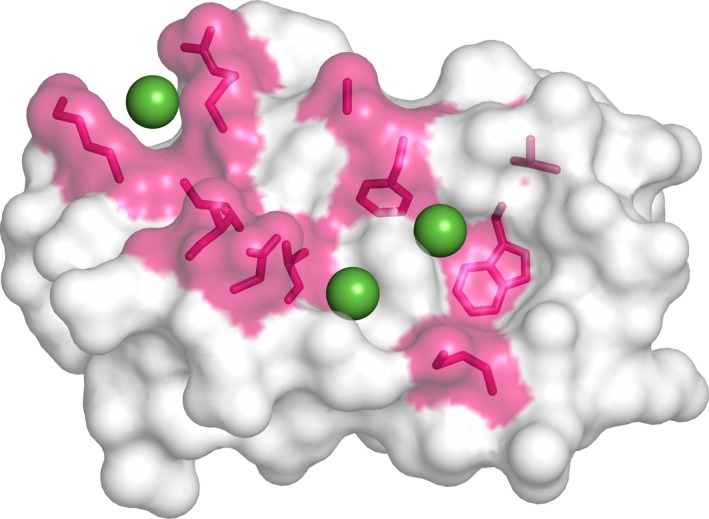
Potential small‐molecule binding pockets in INI1/hSNF5 RPT1. Molecular surface representation of INI1/hSNF5 RPT1 showing the residues that undergo chemical shift more than standard deviation highlighted in magenta, and in green spheres representing three potential small‐molecule binding pockets that were identified by the LIGSITE program.

The highly context dependent functionalities of the SWI/SNF complex make it very difficult to use genetic approaches to determine the role of the interaction between MYC and the INI1. The use of chemical probes 40 could instead be a more productive approach because their effects are tunable, reversible, and most relevant to this interaction, conditional as they can be introduced at any point during cancer development. The MYC binding region in INI1 RPT1 possesses features potentially amenable for small‐molecule binding, and an *in silico* assessment using LIGSITE 41, which is widely used to evaluate the suitability of proteins to bind small molecules, predicted both the hydrophobic pockets within the MYC binding region as potential small‐molecule binding sites (Fig. 10). Furthermore, targeting domains structurally similar to the INI1 RPT1 has been successfully carried out by fragment‐based screening 42. The biophysical data that we have obtained on INI1 RPT1 will allow the use of both structure‐based design and fragment‐based approaches 43. This strategy could have a dramatic effect on the overall functionality of the SWI/SNF complex, not just on the interaction with MYC. This, though, could provide a means of targeting the SWI/SNF complex itself for therapeutic intervention 15.

MYC is an intrinsically disordered protein where the C‐terminal only folds into a HLH‐Zip structural motif upon binding to the obligatory partner MAX 44. Because of their conformational flexibility, disordered proteins such as MYC 45 typically make multiple interactions with their partner proteins: this multivalency allows them to mediate the formation of stable and dynamic complexes 46. MYC has been reported to interact with multiple subunits of the SWI/SNF chromatin remodeling complex 9, and may bind to other regions of INI1 (i.e., RPT2) 22, although these interactions have not been characterized. This suggests that MYC makes multivalent interactions with the SWI/SNF complex. The *K*_d_ value of the MYC:INI1 RPT1 interaction fits well with a multivalent mode of interaction in which individual components have a relatively modest affinity, but high specificity. In this context, it is relevant to highlight that the only other well‐characterized MYC interaction 47 with a component of chromatin regulatory complexes (i.e., WDR5) has a similar *K*_d_ value to the one for the MYC:INI1 interaction herein reported. A multivalent mode of binding may allow additional levels of regulation (e.g., post‐translational modification) to play a role in directing what type of SWI/SNF:MYC interaction takes place on target genes. Interestingly, the binding regions identified in both MYC and INI1 RPT1 are subjected to post‐translational modifications, such as phosphorylation, acetylation, and ubiquitination 48.

One striking feature of the binding of INI1 on the MYC is its interplay with the binding of the MYC:MAX dimer to DNA. The interaction maps to a large region involving the HLH motif and the beginning of the basic region. This shows that this region is not only involved in dimerization and DNA binding, but it is also important in mediating PPIs. In fact, our study shows for the first time that the interaction of MYC with a cofactor competes with the MYC:MAX dimer binding to DNA. In particular, we have found that binding of INI1 and DNA to the MYC:MAX complex are mutually exclusive: the interaction of INI1 RPT1 with the MYC:MAX dimer does not take place when MYC is bound to E‐box DNA, which is at first hand an unexpected result as conventionally one would expect that a transcription factor would recruit the SWI/SNF complex to its cognate DNA. This has potentially important functional implications. The factors governing the distribution of MYC on the genome are known to be diverse, and to include^49^, ^50^: direct interactions with DNA containing both high‐affinity canonical E‐box sequences and other lower affinity sites, as well as multiple low‐affinity PPIs between the MYC:MAX dimer and other chromatin‐associated protein complexes, and/or transcription factors, such as MIZ1 51, which themselves can bind to DNA. Site distribution has been suggested to depend on the level of MYC in the cell, with lower affinity sites being occupied in tumors with high levels of MYC expression. At sites where MYC:MAX does not directly bind to DNA, the MYC:MAX complex will be available to interact with INI1. At other sites the binding of INI1 to MYC:MAX would compete with DNA with the outcome depending on the relative affinities. As mentioned above, it is possible that MYC makes other contacts with the SWI/SNF complex; the avidity effect of this multivalent mode of binding would then result in a higher affinity interaction, thus enabling the SWI/SNF complex to displace MYC from DNA – especially at lower affinity sites 50. This could explain the observations that the SWI/SNF complex can decrease MYC's binding to some of its target genes in lung cancer cell lines; and that when INI1 is introduced into INI1‐deficient rhabdoid tumor cells, MYC binding is reduced at some of its target genes 22. Our findings about the competition with DNA binding would be, therefore, consistent with INI1 negatively regulating MYC transcriptional activity.

Overall, this work highlights how MYC operates *via* a complex network of competing interactions, and offers a potential means to manipulate its functions.

## Materials and methods

### Material

Chemicals were acquired from Sigma‐Aldrich (St. Louis, MO, USA) or Fisher Scientific (Fremont, CA, USA) and were used without further purification. Ni‐NTA resin was from Qiagen (Manchester, UK), Amicon centrifugal units were obtained from Millipore (Billerica, MA, USA). PCR primers were obtained from IDT (Integrated DNA Technologies, Leuven, Belgium).

### DNA cloning, and protein expression and purification

#### INI1/hSNF5

The DNA encoding the residues 184–258 of INI1 was amplified from human cDNA by PCR and cloned into a modified pRSETA (Invitrogen, Life Sciences, Paisley, UK) expression vector that produces proteins fused to N‐terminally His_6_‐tagged lipoyl domain of *Bacillus stearothermophilus* dihydrolipoamide acetyltransferase. The resulting plasmids were transformed into *E. coli* C41 (DE3) cells. Cells were grown in 2XTY media at 37 °C to mid‐log phase and induced with 1 mm IPTG. The temperature was reduced to 22 °C, and the cells were grown for a further 16 h. Isotopically labeled domains were prepared by growing cells in K‐MOPS minimal media containing ^15^NH_4_Cl and/or [^13^C]‐glucose. Cells where lysed by sonication, and the fusion protein was purified by Ni^2+^‐NTA affinity chromatography. The purified protein was dialyzed overnight in the presence of TEV protease, which cleaves the fusion protein after the lipoyl domain. A second Ni^2+^‐NTA affinity chromatography step was carried out to remove the lipoyl domain and the protein was further purified by gel filtration using a HiLoad 26/60 Superdex 75 column (GE Healthcare, Little Chalfont, UK).

#### c‐MYC:MAX bHLHZip (MYC = 352–437, MAX = 22–102) and c‐MYC:MAX HLHZip (MYC = 368–437, MAX = 36–102)

Soluble His6 MYC:MAX bHLHZip, or His6 MYC:MAX HLHzip heterodimer, was produced in *E. coli* using a pET28a derived plasmid that directs the coexpression of both proteins from a polycistronic mRNA, using a similar approach to Fieber *et al*. 52. Chemically competent *E. coli* BL21 (DE3) cells were transformed with this plasmid. Cells were plated on Luria‐Bertani agar supplemented with kanamycin. A single colony was used to inoculate a culture of either 2XTY broth or K‐MOPS minimal media prepared in D_2_O containing ^15^NH_4_Cl and [^13^C]‐glucose. Cell were grown at 22 °C to an OD 600 of 0.8 and then induced with 1 mm IPTG. Cells were grown for a further 16 h before being collected by centrifugation. Cells were lysed by sonication. For the MYC:MAX bHLHZip complex, this is followed by the addition of DNAse I (150 μL for 1 g of culture pellet) and incubation for 1 h at 37 °C (90 r.p.m.). Centrifugation at 38 000 ***g*** at 4 °C for 45 min is then carried out.

The dimer was purified at 25 °C by affinity chromatography using a HisTRAP column (GE Healthcare) according to the standard protocol for Ni^2+^‐NTA affinity chromatography recommended by the manufacturer, and dialyzed against PBS (with 1–10 mm DTT) overnight at 5 °C. Aliquots of the protein complex were snap‐frozen in liquid nitrogen and stored at −80 °C.

### NMR

INI1/hSNF5 samples prepared for NMR spectroscopy experiments were typically 1.5 mm for structural determination in 90% H_2_O, 10% D_2_O, containing 20 mm potassium phosphate, pH 6.5, 100 mm NaCl, and 5 mm β‐mercaptoethanol. All spectra were acquired using a Bruker DRX800, DRX600, or DMX500 spectrometer equipped with pulsed field gradient triple resonance at 25 °C, and referenced relative to external sodium 2,2‐dimethyl‐2‐silapentane‐5‐sulfonate for proton and carbon signals, or liquid ammonia for that of nitrogen. Assignments were obtained using standard NMR methods using ^13^C,^15^N‐labeled, ^15^N‐labeled, 10%^13^C‐labeled, and unlabeled protein samples 53. Backbone assignments were obtained using the following standard set of 2D and 3D heteronuclear spectra: ^1^H‐^15^N HSQC, HNCACB, CBCA(CO)NH, HNCACO, HNCO, HBHA(CO)NH, and ^1^H‐^13^C HSQC. Additional assignments were made using 2D TOCSY and DQF‐COSY spectra. A set of distance constraints were derived from 2D NOESY spectra recorded from a 1.5 mm sample with a mixing time of 120 ms. Hydrogen bond constraints were included for a number of backbone amide protons whose signals were still detected after 10 min in a 2D ^1^H‐^15^N‐HSQC spectrum recorded in D_2_O at 278* *K (pH 5.0). Candidates for the acceptors were identified using the program hbplus for the hydrogen bond donors that were identified by the H–D exchange experiments. When two or more candidates of acceptors were found for the same donor in different structures, the most frequently occurring candidate was selected. For hydrogen bond partners, two distance constraints were used where the distance ^(D)^H–O^(A)^ corresponded to 1.5–2.5 Å and ^(D)^N–O^(A)^ to 2.5–3.5 Å. Torsional angle constraints were obtained from an analysis of C’, N, C_α_ H_α_ and C_β_ chemical shifts using the program talos 54. The stereospecific assignments of H_β_ resonances determined from DQF‐COSY and HNHB spectra were confirmed by analyzing the initial ensemble of structures. Stereospecific assignments of H_γ_ and H_δ_ resonances of Val and Leu residues, respectively, were assigned using a fractionally ^13^C‐labeled protein sample 55. The three‐dimensional structure of the INI1 domain (residues 183–258) was calculated using the standard torsion angle dynamics‐simulated annealing protocol in the program cns 1.2 56. Structures were accepted where no distance violation was greater than 0.25 Å and no dihedral angle violations > 5°. The final coordinates have been deposited in the Protein Data Bank (PDB accession no. 57LB).

Labeled MYC:MAX samples prepared for NMR spectroscopy experiments were typically 200–300 μm in PBS, 10% D_2_O, pH 7, and 1 mm DTT. All spectra were acquired using a Bruker DRX800 (Bruker BioSpin, Billerica, MA, USA) (binding experiments) or DRX950 (for NMR assignments) spectrometers equipped with pulsed field gradient triple resonance cryoprobe at 25 °C. Backbone assignments were carried using the following standard set of 3D heteronuclear spectra on deuterated samples: HNCO, HN(CA)CO, HNCACB, CBCA(CO)HN. The assignments have been deposited in the Biological Magnetic Resonance Bank (BMRB, accession number 27571).

Chemical shift perturbations were calculated using the root mean square deviation of the changes of the H and N chemical shifts, using a correction factor for the N chemical shifts as discussed by Williamson 57.

### Crystallography

Crystals of INI1/hSNF5 (concentration of 6 mg·mL^−1^) were grown using the vapor diffusion method at 4 °C using a precipitant of 1.4 m tri‐sodium citrate and 100 mm HEPES pH 7.5. Crystals were immersed into the precipitant solution supplemented with 20% (v/v) glycerol prior to vitrification by direct immersion into liquid nitrogen. Native data was collected in‐house on an Fr‐E Superbright rotating anode generator (Rigaku Corp, Tokyo, Japan), equipped with a MarDTB image plate detector (marresearch GmbH, Norderstedt, Germany). Diffraction data were indexed and integrated with xds 58 and scaled and merged with scala [ 59]. The crystal structure of the truncated construct (183–252) of human INI1 was solved by molecular replacement using the NMR structure as a search model. Density modification produced experimental maps that allowed for manually refinement using main and coot 60. The crystal structure was refined to 2.1 Å using phenix 61 and is consistent with the NMR structure (RMSD of 0.77 Å for the backbone atoms of residues 185–245). The crystallographic data are summarized in Table 2. The validity of all models was routinely determined using molprobity (http://molprobity.biochem.duke.edu/) and by using the free *R* factor to monitor improvements during building and crystallographic refinement. The final coordinates have been deposited in the Protein Data Bank (PDB accession no. 57LA).

## Accession numbers

NMR structure of INI1/hSNF5 RPT1‐ PDB ID: 5L7B. Crystal structure of INI1/hSNF5 RPT1‐ PDB ID: 5L7A. NMR assignments of c‐MYC:MAX bHLHZip complex – BMRB accession number 27571.

## Conflict of interest

The authors declare that they have no conflict of interest.

## Author contributions

GZ conceived and supervised the study; MDA, NH, MB, and GZ performed experiments; SS, MDA, MB, and GZ carried out data analysis; GZ and MB wrote the manuscript with contributions from all authors.
